# Berberine isolation from *Coscinium fenestratum*: optical, electrochemical, and computational studies†

**DOI:** 10.1039/d3ra01769a

**Published:** 2023-06-07

**Authors:** R. M. Gamini Rajapakse, Benjamin R. Horrocks, A. U. Malikaramage, H. M. N. P. Gunarathna, M. G. S. A. M. E. W. D. D. K. Egodawele, J. M. Susanthi Jayasinghe, Udayana Ranatunga, W. H. M. R. N. K. Herath, Lahiru Sandakelum, Shane Wylie, P. G. P. R. Abewardana, V. N. Seneviratne, L. L. K. Perera, D. Velauthapillai

**Affiliations:** a Department of Chemistry, Faculty of Science, University of Peradeniya Peradeniya 20400 Sri Lanka rmgr1521961@gmail.com; b School of Natural and Environmental Sciences, Newcastle University Newcastle Upon Tyne NE1 4LB UK ben.horrocks@newcastle.ac.uk; c Advanced Nanomaterials for Clean Energy and Health Applications, Faculty of Engineering and Science, Western Norway University of Applied Sciences Campus Bergen, Kronstad Bergen D412 Norway

## Abstract

Berberine was extracted from *Coscinium fenestratum* (tree turmeric) and purified by column chromatography. The UV-Vis absorption spectroscopy of berberine was studied in acetonitrile and aqueous media. TD-DFT calculations employing the B3LYP functional were found to reproduce the general features of the absorption and emission spectra correctly. The electronic transitions to the first and second excited singlet states involve a transfer of electron density from the electron donating methylenedioxy phenyl ring to the electron accepting isoquinolium moiety. An estimate of the electrochemical gap (2.64 V) was obtained from microelectrode voltammetry and good agreement was found with quantum chemical calculations using the cc-pVTZ basis set and the B3LYP, CAM-B3LYP and wB97XD functionals. The calculations indicate spin density of the radical dication is delocalised over the molecule. These basic data are useful for assessment of the synthesis of donor–acceptor polymeric materials employing oxidative polymerization or co-polymerisation of berberine.

## Abstract 2

Berberine is isolated from tree turmeric and its optical and electrochemical properties interpreted using quantum chemical simulation.

## Graphical abstract

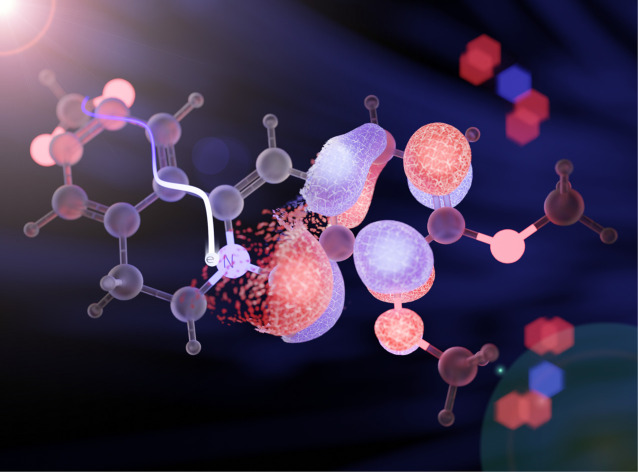

## Introduction

Obtaining raw materials for technological applications from sustainable resources is one important aspect of responsible production and the use of renewable feedstocks can contribute to the 12^th^ sustainable development goal (SDG 12).^1–3^ In electronics, extensive use of non-renewable, non-biodegradable, and potentially toxic materials, such as GaAs, has created a severe solid waste burden. Materials extracted from biological sources, such as plants, are already of significant interest for future electronics.^4,5^ Organic materials, such as electronically conducting polymers (ECPs), are increasingly used as antistatic materials, organic solar cells, printed electronic circuits, organic light-emitting diodes, actuators, electrochromic devices, supercapacitors, chemical- and bio-sensor arrays.^6^ ECPs are usually synthesized by chemical- or electro-polymerization of conjugated organic molecules and therefore plant-based sources of suitable monomers are highly desirable. 3^rd^ generation ECPs, known as donor–acceptor (D–A) polymers, are those which contain electron-rich D and electron-deficient A moieties within the same molecule or copolymers containing electron-rich D and electron-deficient A monomers. Such D–A polymers are of particular interest because they have the potential for intrinsic conductivity in the undoped state by virtue of electron transfer between D and A moieties.^7–11^

**Fig. 1:**
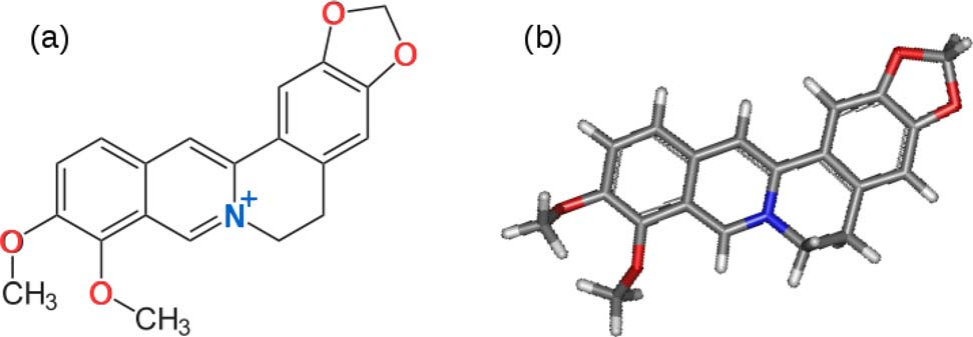
(a) The chemical structure of the berberine cation. The electron donating methylenedioxy and methoxy groups are shown with oxygen atoms in red and the electron accepting isoquinolium moiety is shown with the nitrogen atom in blue. (b) Optimised 3D structure obtained from a calculation using the B3LYP/cc-pVTZ model chemistry.

Berberine and its further *O*-methylated forms, which exist as salts, belong to the family of isoquinolinium alkaloids, which are medicinally active components. They are present in the stems, roots and barks of many tropical plants such as *Berberis aristata* (turmeric)^12^*Berberis vulgaris* (barberry),^13^*Hydrastis canadensis* (goldenseal),^14^ and *Coscinium fenestratum* (tree turmeric).^15^ It has been reported that there are about 400–450 species of the genus *Berberis* belonging to the Berberidaceae family which contain berberine.^16^ Medicinal and therapeutic applications of berberine have been extensively investigated,^17–21^ but its use as a monomer to synthesize donor–acceptor (D–A) ECPs is yet to be explored. Berberine cation has an extended conjugated aromatic π-electron system with an electron-deficient isoquinolinium moiety and electron-rich phenyl segments due to the methylenedioxy and methoxy groups present in the structure shown in Fig. 1.

The electron-deficient isoquinolinium ion attracts electrons of the π-system and acts as an acceptor (A) moiety. The electron-rich methylenedioxy and methoxy groups donate electrons to the phenyl segments to which these groups are attached making these phenyl segments electron-rich enabling them to act as electron donors (Ds). As such, the berberine cation is an example of a D–A monomer present in natural products. The presence of highly polar groups makes the material soluble in polar solvents such as water, acetonitrile and dimethyl sulfoxide (DMSO). This suggests it would be possible to use the electro-polymerisation strategy previously employed to prepare D–A polymers from synthetic conjugated monomers.^7,22–26^ First, it is necessary to investigate the electronic and electrochemical properties of berberine cation.

In this report, we employ quantum chemical simulations of berberine to interpret its spectroscopic, electronic, structural, and electrochemical properties. Berberine was extracted from *Coscinium fenestratum* (tree turmeric) and isolated using chromatographic techniques. The pure isolate was characterized by ^1^H-NMR and UV-visible absorption spectroscopy. The optical spectroscopy and electrode potentials of the reduction and oxidation reactions of berberine are then compared with density functional calculations.

## Experimental and computational methods

### Materials

All solvents were distilled prior to use. Thin Layer Chromatography (TLC) was run using silica gel 60 F_254_ MERCK on aluminum sheets and was visualized with anisaldehyde reagent [anisaldehyde : conc. H_2_SO_4_ : glacial acetic acid : H_2_O − 2 : 3 : 40 : 50] and Dragendorff reagent [a mixture of equal parts (v/v) of 1.7% bismuth subnitrate in 20% acetic acid in water and 40% potassium iodide solution]. Flash chromatography was performed using aluminum oxide, neutral activity I–II for column chromatography (Merck). A commercial sample of berberine chloride (Sigma Aldrich, UK) was used in some experiments (fluorescence and infrared spectroscopy, microelectrode voltammetry, Beer's law tests). *Coscinium fenestratum* stem was obtained from Madeniya, Kithulgala, Central Province, Sri Lanka.

### Quantum chemical methods

Computer simulations of the electrochemical and spectroscopic properties of berberine cation were performed using Gaussian 09 software.^27^ Gabedit was used to aid interpretation of the calculation output.^28^ The optimized geometries of the berberine cation in vacuum, water, and acetonitrile were computed using several functionals (B3LYP, CAM-B3LYP, M11 and wB97XD). Solvation was treated by the implicit polarizable continuum model (PCM)^29^ in the variant implemented as the default in Gaussian 09. Optical spectra were calculated using time-dependent DFT (TD-DFT) to determine vertical excitation energies and then convoluted with Lorentzian functions of 15 nm half-width. Calculations of ^1^H-NMR spectra employed the gauge-independent atomic orbital (GIAO) method.

### UV-visible spectroscopy

Absorption spectra in acetonitrile and aqueous solution are reported as molar absorptivity. The spectra were recorded on a Shimadzu UV-1800 (serial no: A11635305394 CD) spectrometer. Spectra over a range of concentrations (from about 0.4 mM to 25 μM) to confirm the applicability of Beer's law were recorded on a Cary 60 (serial no. MY21289228) spectrometer.

### Fluorescence spectroscopy

Fluorescence excitation–emission maps were recorded on a Shimadzu RF6000 fluorescence spectrometer (serial no. A40245801879). The cuvette was quartz with a standard 1 cm path length and the data reported is corrected for lamp power and detector sensitivity by the manufacturer-supplied software.

### FTIR spectroscopy

Infrared (FTIR) spectra were recorded on a Shimadzu IRAffinity-1S spectrometer (serial no. A21965100120) using an ATR configuration. The resolution was 4 cm^−1^ and 64 scans were co-added and averaged.

### Electron microscopy

Electron micrographs and energy dispersive X-ray spectra (EDX) of solid samples of berberine isolate were obtained using an FEI Quanta 3D FEGSEM.

### Electrochemistry

Slow scan rate cyclic voltammetry studies of berberine were carried out using a Metrohm PGSTAT204 (serial no: AUT50184) electrochemical workstation. The electrochemical studies were conducted in acetonitrile solution containing tetrabutylammonium hexafluorophosphate (0.1 M) and berberine (3 mM in acetonitrile). The solution was purged with dry nitrogen gas for 20 min before the experiment. Flow of nitrogen gas was passed above the solution during the application of the potential program. The working, reference and counter electrodes used were glassy carbon discs, Ag/AgCl (3.0 mol dm^−3^ KCl(aq.)), and platinum gauze, respectively.

Microelectrode voltammetry was performed using a 33 μm diameter carbon microelectrode (ALS, Cat No. 002002, Japan) and a PalmSens3 potentiostat (serial no. PS314D116i). The counter electrode was a Pt wire, and the reference was an aqueous Ag/AgCl (3 M KCl) electrode. For high scan rate experiments, an AgQRE was connected in parallel to the reference; this reduces the impedance of the circuit and improves the data quality at high frequencies without disturbing the reference potential. The cell was sparged with dry nitrogen gas for about 20 min prior to the measurements.

### NMR spectroscopy

The ^1^H-NMR spectra were recorded using an Xpulse HFX Oxford Instruments (serial no: XMAG-052) spectrometer with a base operating frequency of 400 MHz. The solvent was CD_3_OD (Sigma Aldrich, USA) and the chemical shift standard was tetramethylsilane (Sigma Aldrich, USA)

### Isolation and extraction of berberine from *Coscinium fenestratum*

**Fig. 2:**
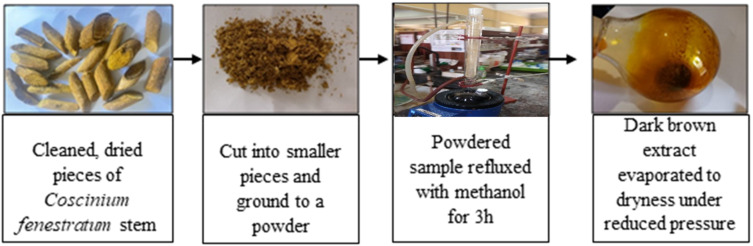
The procedure for extraction of methanol soluble components present in *Coscinium fenestratum*. The dark brown extract after evaporation in the rightmost image is the crude extract discussed in the main text. Further purification was undertaken by column chromatography.

The procedure for extraction of berberine is shown in Fig. 2. The dried crude extract was dissolved in dichloromethane (DCM), mixed with alumina and solvents were evaporated to form a dry powder. Acidified alumina was packed in a column and the solidified crude was introduced using the dry loading method. Gradient elution started with 100% DCM and the polarity was progressively increased using methanol (up to 6% MeOH). The samples were collected into test tubes and analyzed by thin-layer chromatography (TLC).

## Results and discussion

### Isolation of berberine

The methanolic crude product was subjected to thin layer chromatography (TLC) in two solvent systems ((1) 8% methanol : dichloromethane and (2) butanol : ethyl acetate : acetic acid : water in 2.5 : 5 : 1.5 : 1) to identify the compounds present (Fig. 4A). Subsequently, TLC plates from the product after purification by column chromatograph were obtained and these are presented in Fig. 4B. First, we discuss the TLC plates from the crude product. Three compounds were separated according to their polarity in the 8% methanol : dichloromethane (DCM) solvent system as shown in Fig. 4A-1 and their retardation factors (*R*_f_) determined. Three spots are distinguished with anisaldehyde treatment: *R*_f_ = 0.72, blue, compound with low polarity; *R*_f_ = 0.45, yellow; and *R*_f_ = 0.1 dark blue compound with highest polarity. The spot visualized in yellow color was suspected to be berberine. When the TLC was run in the solvent system butanol : ethyl acetate : acetic acid : water in 2.5 : 5 : 1.5 : 1 volume ratio (Fig. 4A-2), Three spots could be seen under UV light, however after spraying with Dragendorff reagent,^30–32^ one dark orange colored spot characteristic of alkaloids is visible. The *R*_f_ value of the dark orange spot is 0.61. This may correspond to one of the alkaloids, such as palmatine, jatrorrhizine, berbamine, aromaline, oxyberberine, or karachine present in *Coscinium fenestratum.*^33–35^

The ^1^H-NMR spectrum in CD_3_OD of the crude product (Fig. 5A) confirms that it contains a mixture of compounds as revealed by the TLC experiments. Additional multiplets between 3.5 and 4.0 ppm and a multiplet between 0.5 and 1.5 ppm, were observed in the ^1^H-NMR spectrum of the crude extract (Fig. 5A, red circles) compared to the ^1^H-NMR spectrum of isolated pure berberine (Fig. 5B). The crude extract contains a mixture of the alkaloids that are present in *Coscinium fenestratum*.

In Fig. 4B, the TLC plate 1 is typical of the first set of fractions separated from the column. Blue colored spots indicate the compounds that separated under the low polarity. Then, in plate 2, both the blue and yellow spots can be seen as the polarity is increased with the increasing fraction of MeOH in the eluent. After increasing the MeOH percentage further, a more polar compound was eluted as shown by the orange spots on the plates 3–6 in Fig. 4B. All of these orange spots have the same *R*_f_ value of 0.45. The fractions that contained this yellow compound with the same *R*_f_ value of 0.45 were therefore pooled (red circles in Fig. 4B) and evaporated to dryness. The ^1^H-NMR spectrum of this isolated compound is shown in Fig. 5B. After increasing the MeOH percentage further (up to 8%), the polar compound with the highest polarity was eluted (Fig. 4B-7).

**Fig. 3:**
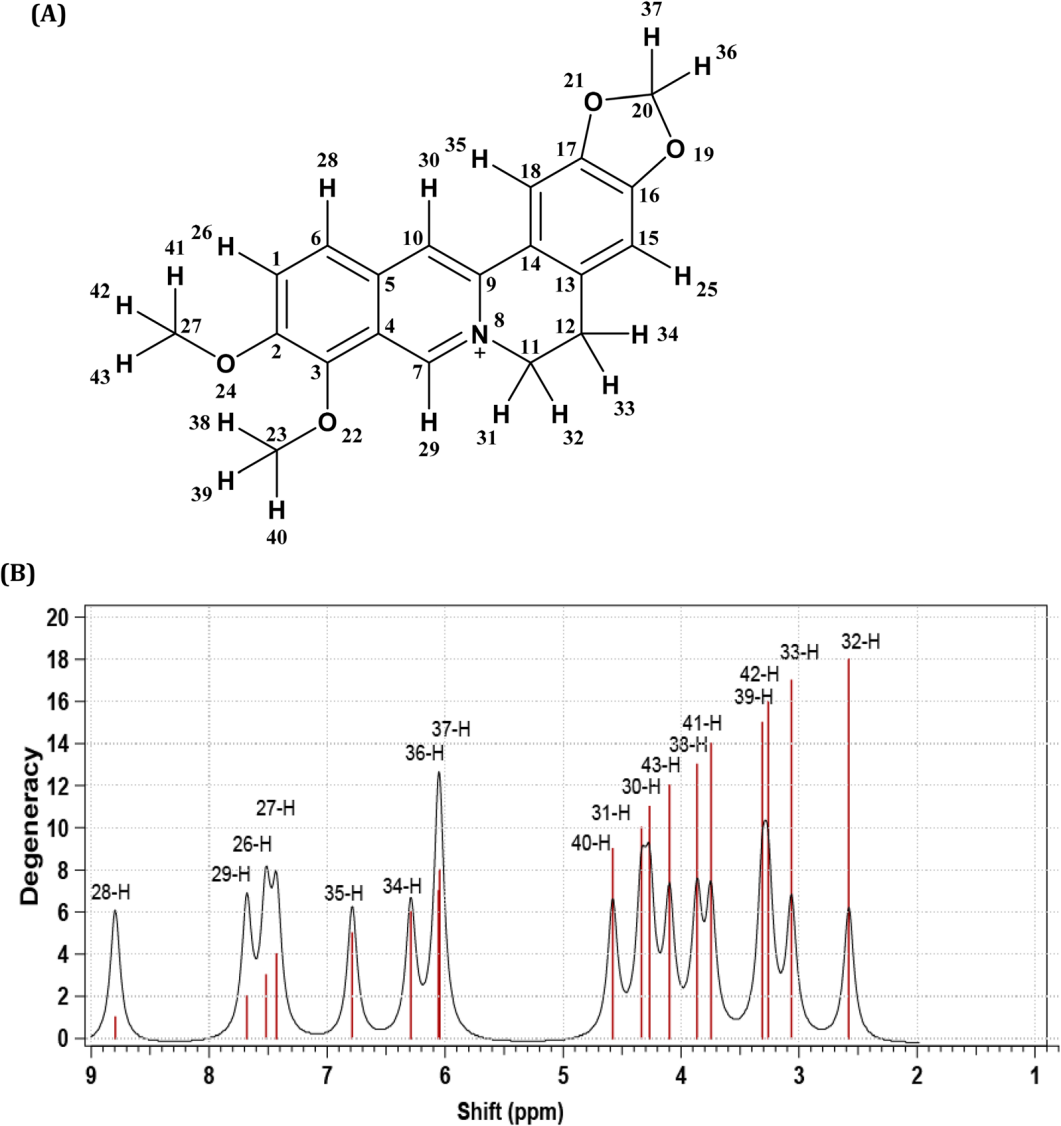
(A) Berberine structure with atom labels. (B) The ^1^H-NMR spectrum of berberine calculated using B3LYP/6-31G with implicit methanol solvation.

**Fig. 4:**
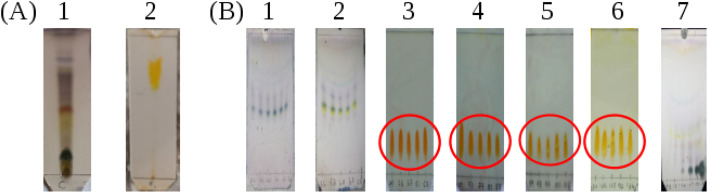
(A) The TLC profiles of methanolic crude product. The two plates correspond to two solvent systems. (A1) 8% methanol : dichloromethane and (A2) butanol : ethyl acetate : acetic acid : water in 2.5 : 5 : 1.5 : 1 volume ratio. (B) The TLC plates from different fractions separated by the column chromatography in methanol/dichloromethane mixtures. Plates (B1) and (B2), eluted using 2.5% methanol : dichloromethane after spraying with anisaldehyde reagent; plates (B3)–(B6) eluted using 3% methanol : dichloromethane after spraying with Dragendorff reagent and plate (B7) eluted using 6% methanol : dichloromethane after spraying with anisaldehyde reagent.

**Fig. 5:**
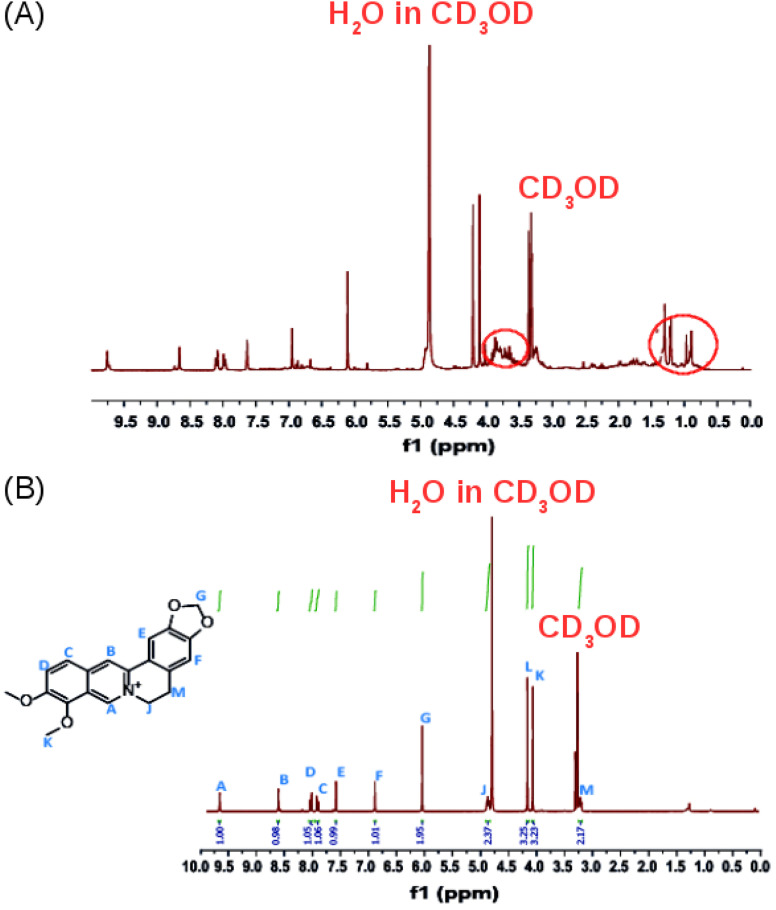
(A) The ^1^H NMR spectrum (400 MHz) of the crude product. (B) The ^1^H-NMR spectrum of the isolated, purified product in CD_3_OD.

The purity of the isolated berberine (pooled from fractions 3 to 6 of Fig. 4B) is established from the observation of a single spot in the TLC and the generation of color with the Dragendorff reagent which indicates the compound is an alkaloid. The melting point range of the isolate was 192–195 °C, close to that (193.5 °C) reported using differential scanning calorimetry.^36^ Further, all the peaks observed in the ^1^H-NMR spectrum (Fig. 5B) match those in the reported spectrum of berberine.^37^ Quantum chemical calculation of proton chemical shifts (Table 1 and Fig. 3B) was used to interpret the ^1^H-NMR spectrum of berberine. The calculations employed B3LYP/6-31G with implicit solvation by methanol. The dielectric constants of deuterated and non-deuterated methanol are almost the same^38^ and therefore employing the dielectric constant of non-deuterated methanol for the solvation calculation is not expected to contribute a significant error. Although the calculated ^1^H-NMR peak shifts are lower than those determined experimentally for almost all protons except H31, mean absolute errors up to 0.4 ppm and maximum absolute errors up to 1.1 ppm have been reported in an automated framework for ^1^H-NMR chemical shift calculations of small organic molecules when geometries are optimized at the B3LYP/6-31G(d) level in chloroform.^39^ Nevertheless, despite these shifts in peak positions, the theoretical and empirical spectra match in terms of number of protons in different electronic environments and their order of appearance in the spectra.

**Table 1:** Peak assignment of the ^1^H-NMR spectrum of the isolated, purified product (Fig. 5B) from column chromatography of crude methanolic extract of *Coscinium fenestratum* and quantum chemical ^1^H-NMR data (Fig. 3B)

Experimental data				Computational data		
Chemical shift/ppm	No. of protons	Description	Assignment	Chemical shift/ppm	Description	Assignment
3.28	2	Triplet (M/H-32, H-33)	–CH_2_–	2.56	Triplet (H-33)	–CH_2_–
				3.06	Triplet (H-32)	
4.13	3	Singlet (L/H-41, H-42, H-43)	O–CH_3_	3.26	Singlet (H-42)	O–CH_3_
				3.75	Singlet (H-41)	
				4.10	Singlet (H-43)	
4.23	3	Singlet (K/H-38, H-39, H-40)	O–CH_3_	3.87	Singlet (H-38)	O–CH_3_
				3.31	Singlet (H-39)	
				4.58	Singlet (H-40)	
4.95	2	Triplet (J/H-30, H-31)	N–CH_2_	4.27	Triplet (H-30)	N–CH_2_
				4.34	Triplet (H-31)	
6.13	2	Singlet (G/H-36, H-37)	O–CH_2_–O	6.05	Singlet (H-37)	O–CH_2_–O
				6.06	Singlet (H-36)	
6.98	1	Singlet (F/H-34)	Aromatic H	6.29	Singlet (H-34)	Aromatic H
7.69	1	Singlet (E/H-35)	Aromatic H	6.79	Singlet (H-35)	Aromatic H
8.02	1	Doublet (D/H-26)	Aromatic H	7.52	Doublet (H-26)	Aromatic H
8.14	1	Doublet (C/H-27)	Aromatic H	7.43	Doublet (H-27)	Aromatic H
8.73	1	Singlet (B/H-29)	Aromatic H	7.68	Singlet (H-29)	Aromatic H
9.79	1	Singlet (A/H-28)	Aromatic H	8.80	Singlet (H-28)	Aromatic H

Further evidence of the purity of the isolated berberine was obtained using FTIR spectroscopy of solid samples, one of isolated material and a second, commercial sample of berberine chloride. Fig. S2 (ESI†) presents these spectra. A comparison of the fingerprint region (Fig. S2(b)†) shows that the two compounds are the same. The modes previously assigned to the aromatic carbon–carbon mode at 1506 cm^−1^ and the modes associated with the quinolinium (C=N^+^) at 1601 cm^−1^ are clearly observed.^40–42^ Electron microscopy and energy dispersive analysis of X-rays on individual crystals of the isolated berberine demonstrated the presence of C, O, N and Cl atoms (Fig. S3 and S4, ESI†). The latter confirms that the counter anion in the isolate is chloride.

### Optical spectroscopy and quantum chemical simulation of molecular and electronic structure

**Fig. 6:**
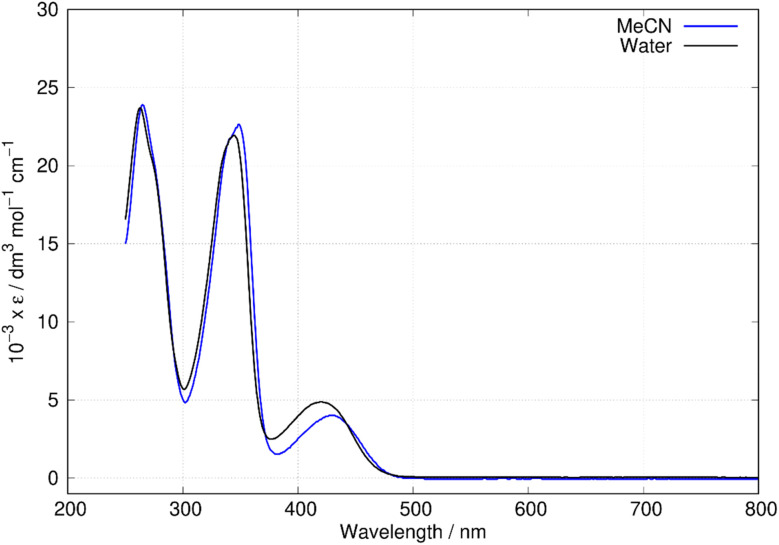
Absorption spectra of berberine in water and acetonitrile. The data is reported as molar absorptivity divided by 10^3^.

Fig. 6 shows the absorption spectra of berberine in aqueous and acetonitrile solutions. Three bands are clearly visible with maxima at *λ* = 430 nm, 349 nm, and 265 nm in acetonitrile. Over the range of concentrations investigated (25 μM–0.4 mM) the absorbance was proportional to concentration, which suggests absence of significant effects due to aggregation or other intermolecular interactions. The measured absorption coefficients (*ε*_430 nm_(CH_3_CN) = 4017 ± 33 dm^3^ mol^−1^ cm^−1^, this work) are like previous reports (*ε*_430 nm_(CH_3_CN) = 4488 dm^3^ mol^−1^ cm^−1^).^43^ The absorption peaks show a small blue-shift in water compared to acetonitrile with maxima at *λ* = 420 nm, 345 nm, and 263 nm. For comparison to the quantum chemical calculations, the vertical excitation energy was estimated from the onset of the absorption spectrum in acetonitrile. A Gaussian function was fitted to the long wavelength side of the peak at 430 nm and the intercept of the tangent of maximum slope with the wavelength axis used to estimate the onset (or equivalently *λ*_max_ + 2*σ*, where *σ* is the fitted standard deviation). This experimental absorption onset of 474 nm corresponds to a photon energy of 2.616 eV.

The nature of the excited states responsible for the optical absorption and emission spectra of berberine was investigated by quantum chemical simulations using time-dependent density functional theory (TD-DFT). Berberine is a rigid molecule and hence the molecular structure at the optimized geometry (Fig. 1b) is hardly different for calculations of the structure in vacuum, water, and acetonitrile. However, the predicted absorption spectrum of berberine cation depends on the environment; the spectrum in vacuum is significantly different from that in the two polar solvents.

**Fig. 7:**
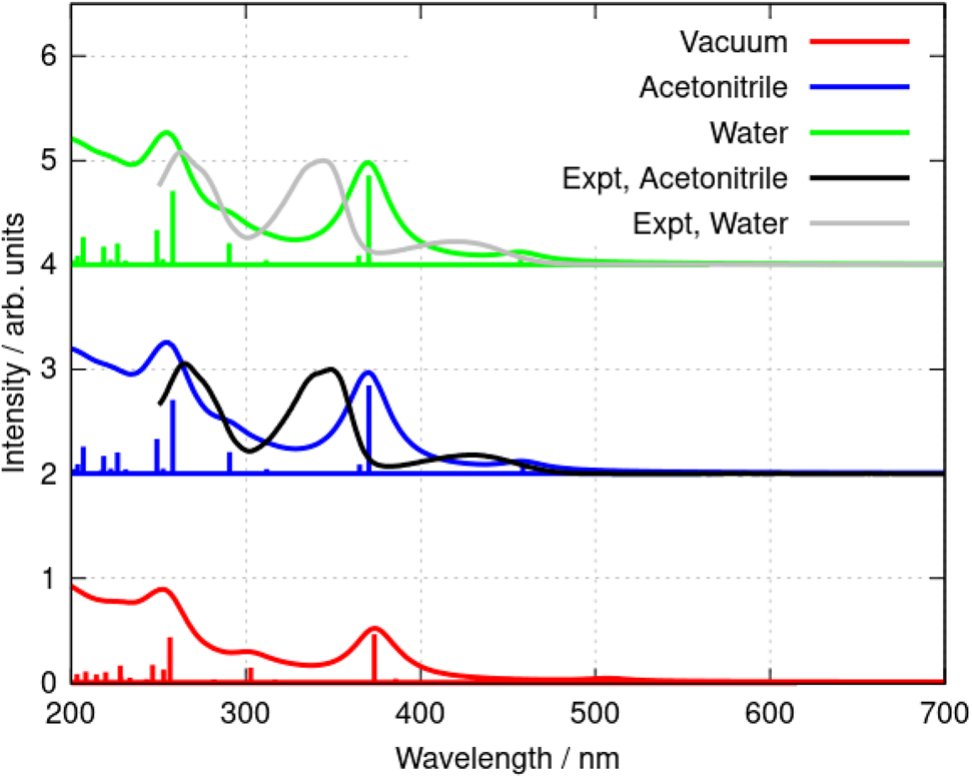
Simulated absorption spectra of the berberine cation in water, acetonitrile, and vacuum. The model chemistry was B3LYP/6-31G(d). The data are offset for clarity and normalised to the peak of the S_0_ → S_2_ transition (near *λ* = 350 nm in the experimental data).

That the inclusion of solvent in the model is essential can be seen clearly in Fig. 7 in which calculated spectra in water, acetonitrile and vacuum are compared (B3LYP/6-31G(d)). In the absence of solvent, the S_1_ state shifts to lower energy and the first absorption peak appears at 508 nm, which is larger than the experimental value of *λ*_max_ = 430 nm in acetonitrile. In acetonitrile and water, the calculated S_0_ → S_1_ transitions are at *λ* = 455 nm and 457 nm respectively; this represents reasonable agreement with the experimental data bearing in mind the limitations of the implicit solvation model^44^ and that the computed values represent vertical transitions without consideration of vibronic effects. However, the small blue-shift of the experimental spectrum in water compared to acetonitrile cannot be reproduced by the DFT methods and implicit solvation models. The TD-DFT calculation also indicates the S_0_ → S_1_ transition is dominated by promotion of an electron from the HOMO to the LUMO (99% contribution).

**Fig. 8:**
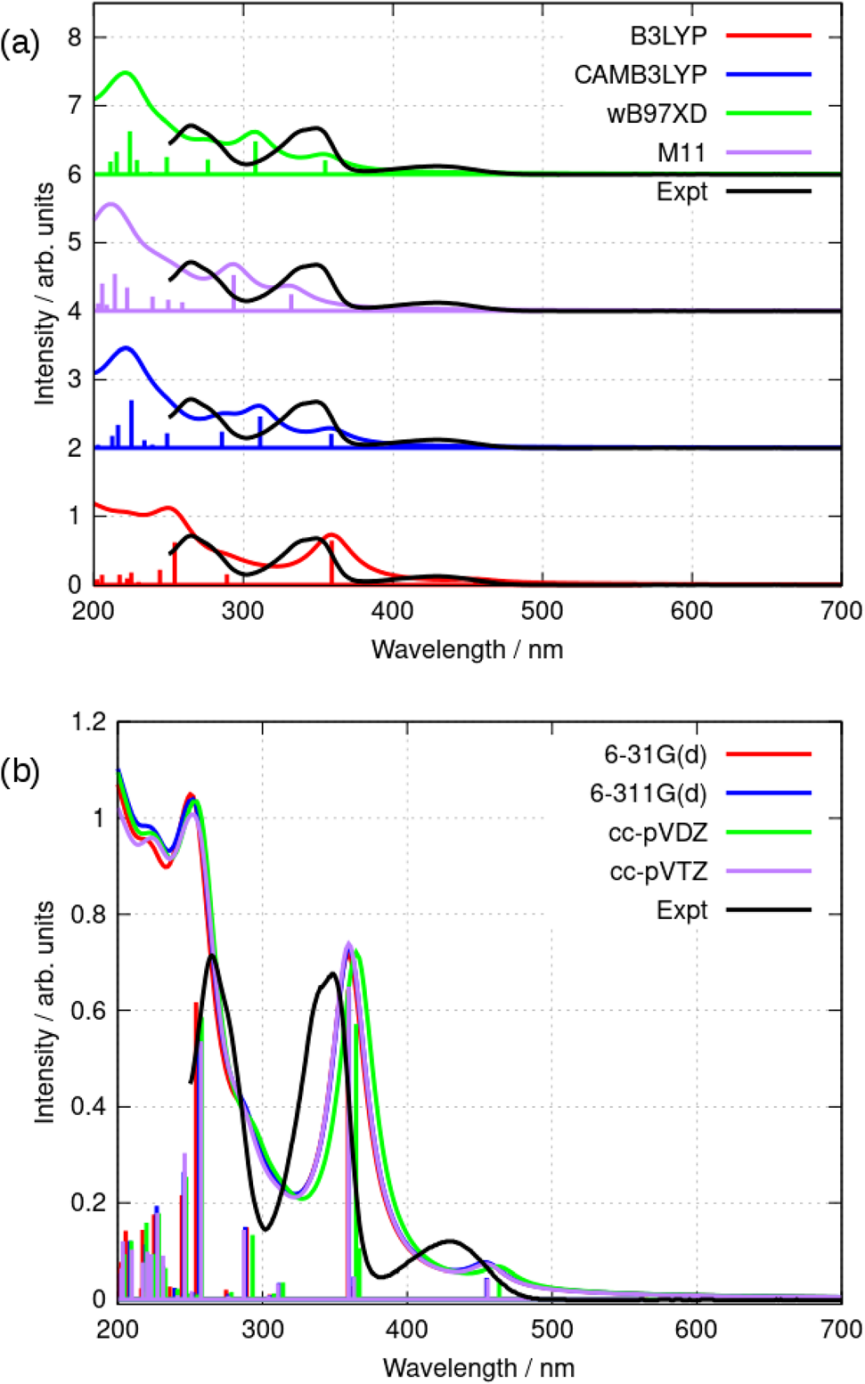
(a) Comparison of the simulated absorption spectra of berberine in acetonitrile using TD-DFT and the PCM solvation model with the experimental data (black). The simulations shown used a selection of functionals and the cc-pVTZ basis set. The simulated curves were obtained using a Lorentzian line shape and a halfwidth of 15 nm in each case. (b) Comparison of the simulated absorption spectra of berberine in acetonitrile using TD-DFT and the PCM solvation model with the experimental data (black). The simulations shown used the B3LYP functional and a series of basis sets. The simulated curves were obtained using a Lorentzian line shape and a halfwidth of 15 nm in each case.

Further simulations were carried out using a selection of functionals and basis sets to test the dependence of the results on the model chemistry. Fig. 8a compares the experimental and simulated absorption spectra in acetonitrile for different choices of density functional. The solvent was modelled throughout by the implicit PCM method using the bulk relative permittivity of acetonitrile. The well-known hybrid functional B3LYP was compared to two range-corrected functionals (CAM-B3LYP^45^ and wB97XD^46^) and the M11 functional,^47^ which has previously been recommended for TD-DFT studies.^48^Fig. 8 shows the vertical excitation energies from each calculation and the simulated spectrum assuming a Lorentzian line shape with half width of 15 nm. The basis-set dependence of the simulated spectra (B3LYP) is shown in Fig. 8b. Only relatively minor differences are observed between the 6-31G(d) and cc-pVTZ basis sets (the largest basis set employed in this work).

As can be seen in Fig. 8, the low-lying excited states for *λ* > 250 nm are satisfactorily modelled by B3LYP whereas the other functionals put the first excited state at *λ* < 400 nm. B3LYP, in contrast, correctly predicts a weak S_0_ → S_1_ transition near 455 nm and a stronger S_0_ → S_2_ transition near 360 nm. The second peak is in fact two near-degenerate transitions comprising major contributions from HOMO − 1 to LUMO and HOMO to LUMO + 1. Tables 2 and 3 summarize the data. The reason for the relatively good performance of B3LYP against the range-corrected functionals is unclear, but similar observations have been made in organic systems.^49,50^

**Table 2:** Computational estimates of the low-lying excited electronic states of berberine in acetonitrile using the B3LYP functional and various basis sets. Values are presented in eV and in nm to facilitate comparison to the spectra

Basis set	*E*(T_1_) − *E*(S_0_)/eV [nm]	*E*(S_1_) − *E*(S_0_)/eV [nm]	*E*(S_2_) − *E*(S_0_)/eV^a^ [nm]
6-31G(d)	2.189 [566]	2.725 [455]	3.424 [362], 3.453 [359]
6-311G(d)	2.203 [563]	2.726 [455]	3.416 [363], 3.447 [360]
cc-pVDZ	2.161 [574]	2.675 [463]	3.379 [367], 3.400 [365]
cc-pVTZ	2.198 [564]	2.722 [456]	3.414 [363], 3.449 [360]
Experiment (*λ*_max_)		2.883 [430]	3.553 [349]

a The calculations indicate two near-degenerate states.

**Table 3:** Computational estimates of the low-lying excited electronic states berberine in acetonitrile using the 6-31G(d) basis set and various functionals. The energy of the lowest lying triplet and singlet states are shown

Basis set	*E*(T_1_) − *E*(S_0_)/eV [nm]	*E*(S_1_) − *E*(S_0_)/eV [nm]
B3LYP	2.189 [566]	2.725 [455]
CAM-B3LYP	2.154 [575]	3.274 [379]
wB97XD	2.234 [555]	3.303 [375]
M11	2.280 [544]	3.470 [357]
Experiment (*λ*_max_)	—	2.883 [430]
Experiment (onset)		2.616 [474]

**Fig. 9:**
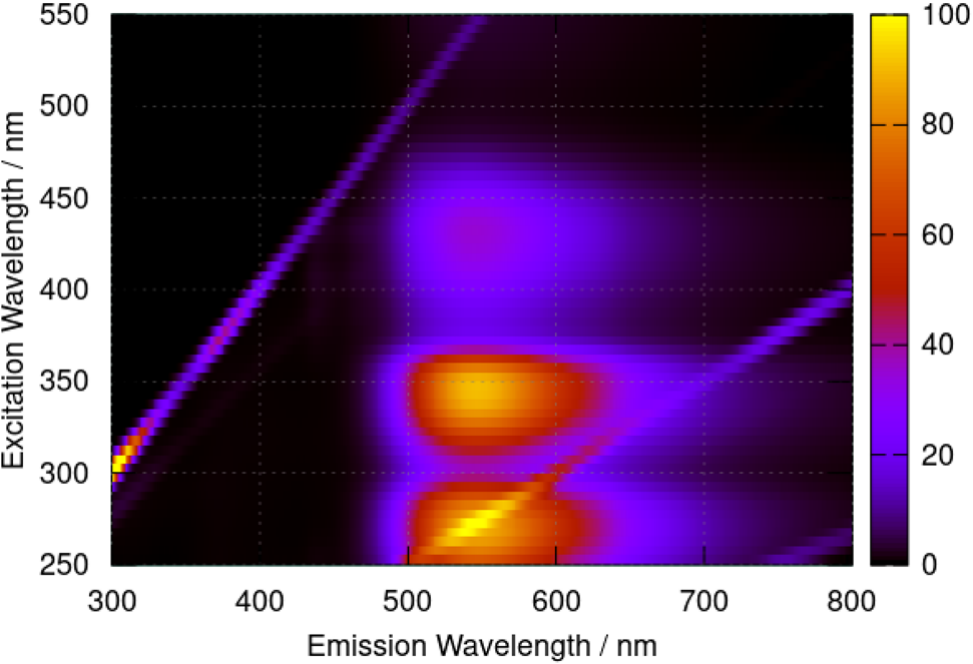
Excitation–emission map for berberine chloride in acetonitrile at a concentration of 40 μM. The colour scale indicates emission intensity in kilocounts.

Fluorescence spectra of berberine cation in acetonitrile show a broad peak with a maximum at 544 nm independent of the excitation wavelength. The excitation–emission map of Fig. 9 shows a simple structure with three maxima at excitation wavelengths of *λ* = 435 nm, 350 nm, and 270 nm which we assign to the transitions observed in the absorption spectrum (Fig. 6) with absorption maxima of *λ* = 430 nm, 349 nm, and 265 nm.

The fluorescence data was modelled by geometry optimisation of the first excited state of berberine cation in acetonitrile using B3LYP/6-31G(d). Both singlet, S_1_ and triplet T_1_ states were optimised in separate calculations. The energies and corresponding wavelengths of the vertical transitions are 2.1760 eV, 570 nm (S_1_) and 1.521 eV, 815 nm (T_1_). The singlet state is clearly the best match to the experimental data, though it should be noted that it has a low oscillator strength *f* = 0.03.

**Fig. 10:**
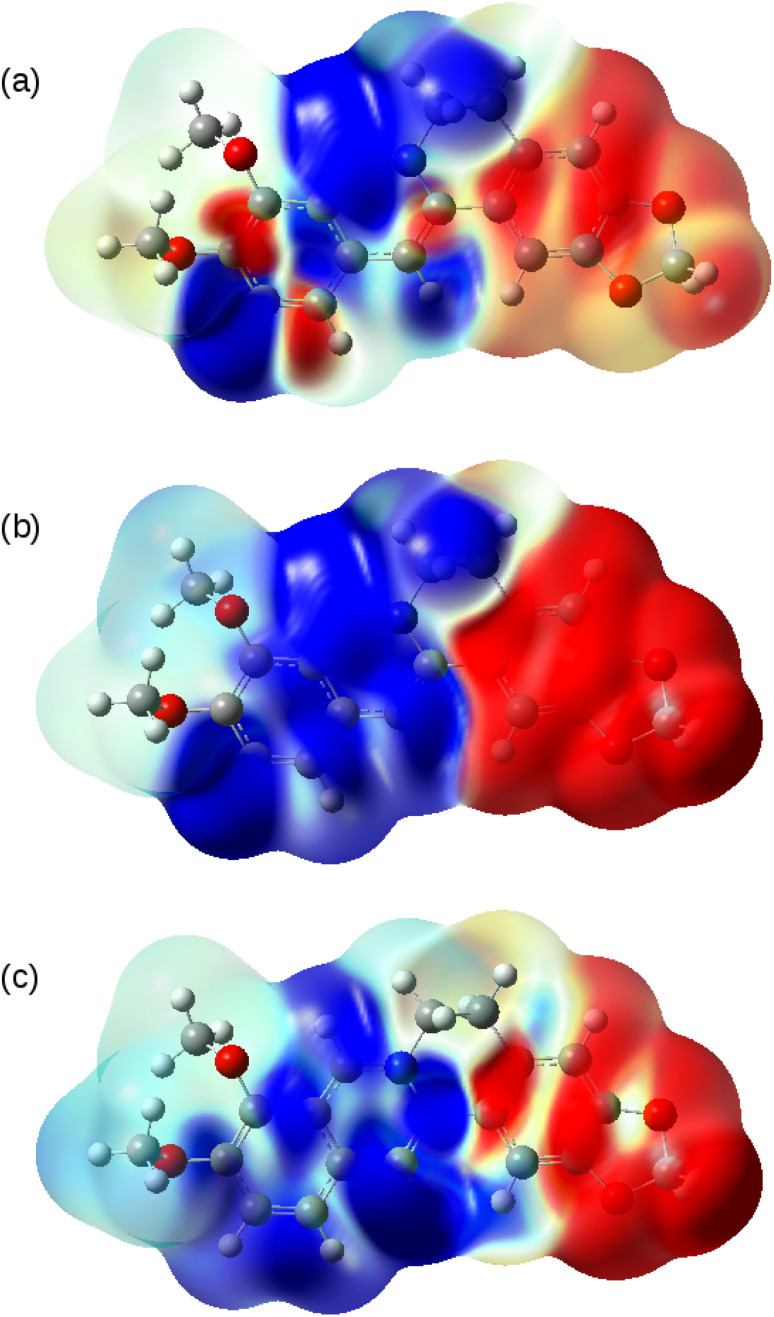
Transition electron density differences mapped onto the electron density of the ground state of berberine at the optimised geometry. Blue indicates gain of electron density and red indicates loss of electron density. The model chemistry was B3LYP/cc-pVTZ with PCM as the solvent model for acetonitrile. (a) S_0_ → T_1_; (b) S_0_ → S_1_ and (c) S_0_ → S_2_.

The nature of the S_0_ → T_1,_ S_0_ → S_1_ and S_0_ → S_2_ transitions are illustrated in Fig. 10. These surfaces show the transition electron density differences mapped onto the electron density of the ground state of the berberine cation in acetonitrile using the B3LYP/cc-pVTZ model. In each case there is a similar pattern with gain of electron density in the electron-accepting isoquinolium moiety and loss of electron density from the electron-donating region of the methylenedioxy groups. In summary, we find that the standard B3LYP functional with the PCM implicit solvent model provides a reasonable description of the optical spectra of berberine in acetonitrile and water solutions. The optical gap is overestimated compared to experiment by about 0.1 eV (Table 3), an error magnitude which is typical of TD-DFT methods and the limitations of implicit solvent models.^44,48^ However, the overall form of the absorption spectrum and the emission spectra are satisfactorily reproduced.

### Electrochemistry and quantum chemical estimation of electrode potentials

**Fig. 11:**
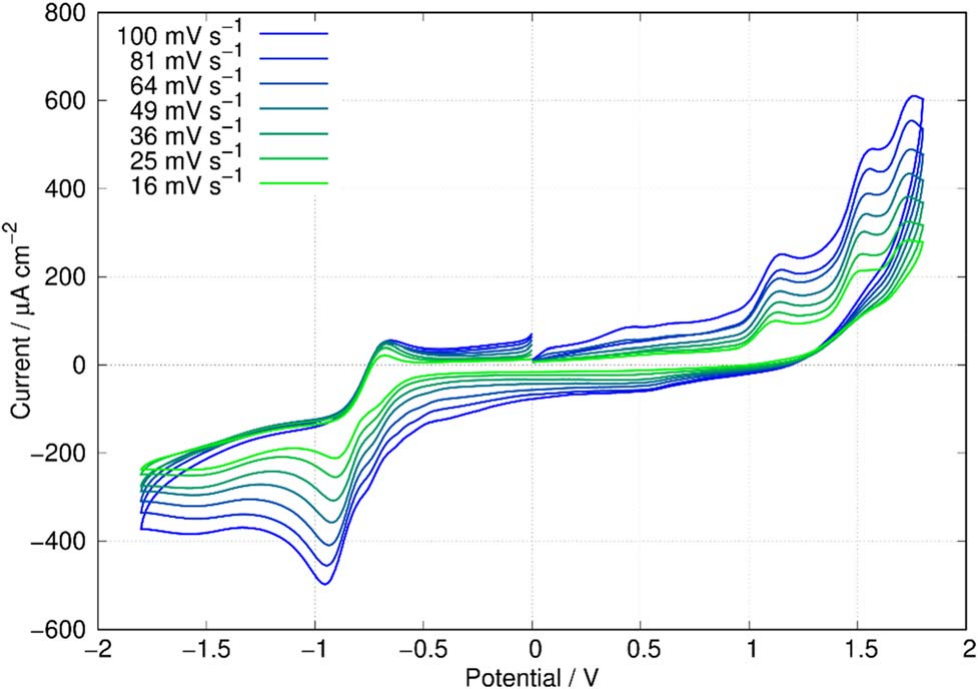
Cyclic voltammetry of berberine in acetonitrile. The working electrode was an 0.5 cm diameter glassy carbon disc, the reference was an aqueous Ag/AgCl (3 M KCl) electrode and the concentration of berberine was 3.0 mM. The solution was sparged with N_2_ to remove dissolved oxygen.

In this report, the electrochemical behaviour of berberine was studied in acetonitrile. The oxidation of berberine in aqueous media takes place at high potentials and leads to the formation of an adsorbed film.^51^ Slow scan cyclic voltammetry of berberine in acetonitrile (Fig. 11) shows a series of chemically irreversible oxidations above +1 V *vs.* aqueous Ag/AgCl. In the negative direction, complex behaviour is observed with two closely spaced waves in the region −0.8 to −1.0 V. In aqueous media, berberine may be reduced to tetrahydroberberine (4e^−^) or in a 2e^−^ process at high pH and there is strong adsorption of the reduction products.^52^ We also observed that repeated scanning of the electrode in the negative potential range in acetonitrile led to fouling of the electrode and the peak separation is much larger than expected for an electrochemically reversible system.

**Fig. 12:**
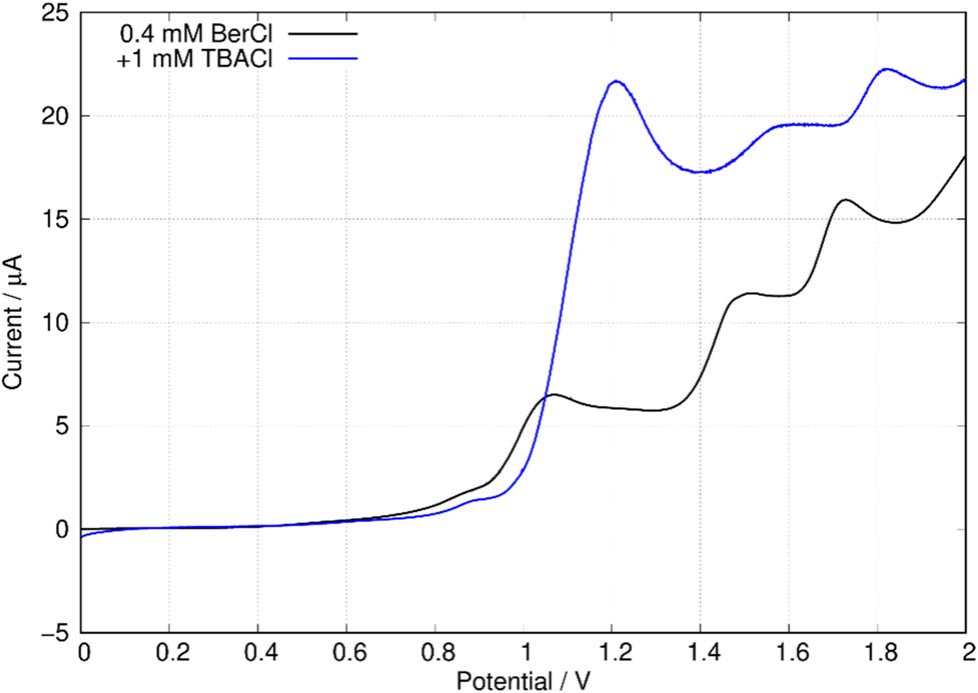
Linear sweep voltammograms of 0.4 mM berberine chloride (commercial sample) in acetonitrile with 0.1 M TBAPF_6_ electrolyte. The scan rate was 0.05 V s^−1^, the working electrode was a 0.3 cm diameter glassy carbon disc, the solution volume was 3 mL, and the reference was an aqueous Ag/AgCl (3 M KCl) electrode. The blue curve shows the effect of the addition of 0.3 mL, 0.01 M TBACl.

In this work, we are principally concerned with estimates of the formal potentials of the oxidation (*E*^2+^˙^/+^) and reduction (*E*^+/0^˙) of berberine for comparison with quantum chemical simulations. First, we compared linear sweep voltammetry of berberine chloride in the presence and absence of TBACl (Fig. 12). The additional chloride increases the anodic peak current of the feature near +1.2 V by a factor of about 3, whilst the peaks at higher potentials are not enhanced. This demonstrates that the first peak is oxidation of chloride anion and confirms that chloride is the counteranion in the isolated samples.

**Fig. 13:**
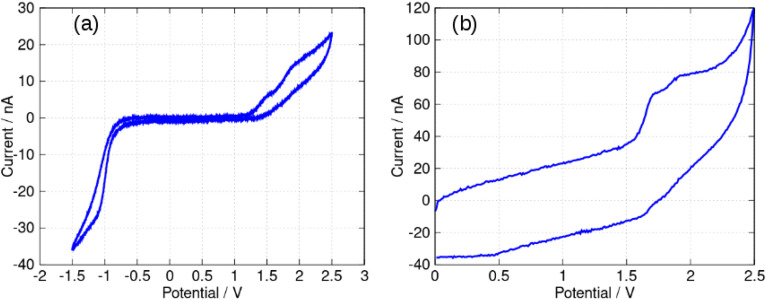
Microelectrode voltammetry of berberine chloride (commercial sample) in acetonitrile. The working electrode was a 33 μm diameter carbon disc. The concentration of berberine was 0.40 mM and the electrolyte was 0.1 M TBAPF_6_. The reference was an aqueous Ag/AgCl (3 M KCl) electrode. The solution was sparged with N_2_ to remove dissolved oxygen. (a) Quasi-steady state voltammetry at a scan rate of 50 mV s^−1^. (b) Fast scan voltammetry at a scan rate of 500 V s^−1^.

In view of the difficulties observed in slow scan cyclic voltammetry, microelectrode measurements were carried out to provide estimates of the electrode potentials for the oxidation (Ber^2+^˙/Ber^+^) and the reduction (Ber^+^/Ber^0^˙) processes. Fig. 13a shows a quasi-steady state microelectrode voltammogram in which the limiting current for the reduction process is about −30 nA and that for the oxidation is approximately 15 nA; this provides support for the assignment of the data in Fig. 11 to (at least) two closely spaced one-electron reductions that are hardly resolved. Below we estimate the formal potential of the reduction, *E*^+/0^˙ from the halfwave potential of the steady-state microelectrode voltammogram as −0.99 V, which is more negative than −0.8 V estimated from the mean of the peak potentials in Fig. 11. This is likely to be due to the difficulties observed in slow scan cyclic voltammetry experiments in this system.

The formal potential of the one-electron oxidation, *E*^2+^˙^/+^, was estimated as 1.66 V from the fast scan CV data of Fig. 13b. The peak separation was close to 100 mV which suggests the electron transfer is quasi-reversible at 500 V s^−1^ and the mechanism is of the E_q_C type. The formal potential is therefore likely to be underestimated from the slow scan cyclic voltammetric experiment because of the effect of rapid loss of the reduction product on the Nernstian equilibrium at the electrode surface at slow scan rates.

Quantum chemical estimates of these electrode potentials were made using the same model chemistries employed in the study of the electronic excited states. The electrode potentials were computed using frequency calculations at the optimised geometry to estimate the free energies of the berberine cation and its one-electron oxidation and reduction products. The PCM model for implicit solvation was employed, but the free energy in solution was still estimated using the treatment of the molecular partition functions based on the ideal gas – no corrections for the condensed phase were applied beyond the inclusion of the solvent dielectric effect in the electronic structure calculation. Absolute potentials were estimated from the difference in free energies and then converted to the reference electrode scale used in the experimental work using a value of 0.210 V for the Ag/AgCl (3 M KCl) *versus* SHE and the recent computational estimates for the absolute potential of the SHE.^53^ The data is summarised in Table 4.

**Table 4:** Computational estimates of the electrode potentials for the one-electron oxidation (*E*^2+/+^) and the one-electron reduction (*E*^2+/+^) of berberine in acetonitrile. The electrochemical gap (Δ*E*) is the difference between these two electrode potentials

Calculation	*E* ^2+^˙^/+^/V	*E* ^+/0^˙/V	Δ*E*/V
B3LYP/6-31G(d)	1.116	−1.241	2.357
B3LYP/6-311G(d)	1.552	−1.071	2.623
B3LYP/cc-pVDZ	1.455	−1.161	2.616
B3LYP/cc-pVTZ	1.536	−1.083	2.618
CAM-B3LYP/cc-pVTZ	1.697	−1.025	2.722
M11/cc-pVTZ	1.899	−0.977	2.865
wB97XD/cc-pVTZ	1.627	−1.055	2.682
Experiment	1.65	−0.99	2.64

Good agreement is observed between the experimental and computed values of the electrode potentials, though it should be noted that (i) there is substantial uncertainty on the experimental value of *E*^+/0^˙, (ii) we have ignored the liquid junction potential at the acetonitrile/water interface and (iii) there is uncertainty on the value of absolute potentials. Nevertheless, some of the systematic errors will cancel when the electrochemical gap (Δ*E* = *E*^2+^˙^/+^ − *E*^+/0^˙) is evaluated and in this case good agreement with the calculations using the cc-pVTZ basis set and the B3LYP, CAM-B3LYP and wB97XD functionals is obtained.

**Fig. 14:**
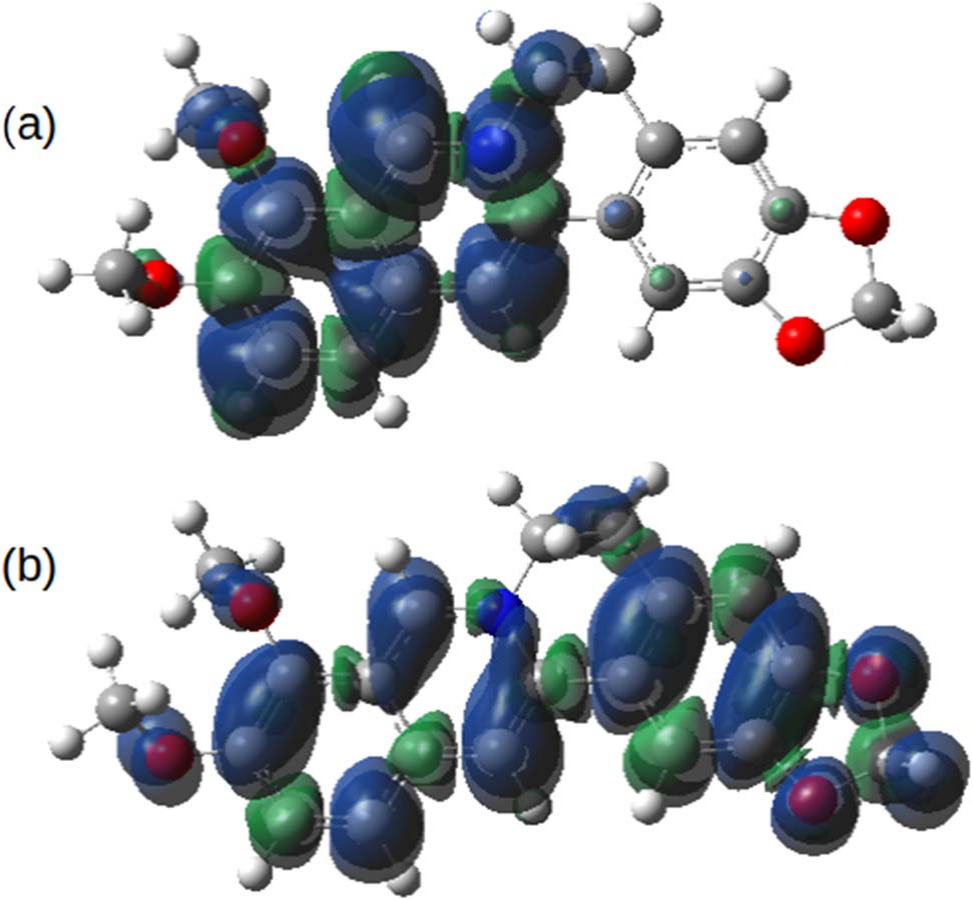
Spin density of berberine calculated at the optimised geometries of (a) the one-electron reduction product, Ber^0^˙ and (b) the one-electron oxidation product, Ber^2+^˙. The model chemistry was B3LYP/cc-pVTZ with implicit solvation by acetonitrile calculated using the PCM model.

Fig. 14 shows the spin density on the one-electron reduction and oxidation products calculated using B3LYP/cc-pVTZ with implicit solvation by acetonitrile. In the case (Fig. 14a) of the reduced form, Ber^0^˙, the spin density is dominantly associated with the isoquinolinium portion of the molecule as might be expected from the electron accepting nature of this group. However, the spin density of Ber^2+^˙ is delocalised over the whole π-system. In principle, therefore, radical-coupling reactions could occur at multiple sites. Copolymerisation of berberine with other monomers used in oxidative electropolymerisation (pyrroles, thiophenes) is likely to lead to a mixture of products.

## Conclusions

Berberine can be isolated in pure form from *Coscinium fenestratum* (tree turmeric) by a straightforward solvent extraction method. The purity and identity of the berberine was confirmed by ^1^H NMR and chemical tests. In agreement with previous reports,^43^ the optical absorption spectrum of berberine cation shows three bands with absorption maxima at *λ* = 430 nm, 349 nm, and 265 nm in acetonitrile. TD-DFT calculations of the vertical excitation energies using the well-known B3LYP functional give satisfactory agreement with the experimental spectra. The calculations indicate the peak at 430 nm corresponds to an S_0_ → S_1_ transition dominated (99%) by the HOMO–LUMO transition. The second peak near 349 nm is in fact two near-degenerate transitions comprising major contributions from HOMO − 1 to LUMO and HOMO to LUMO + 1. Both S_0_ → S_1_ and S_0_ → S_2_ transitions involve a gain of electron density in the isoquinolium portion of the molecule and a loss of electron density from the phenyl methylene dioxy ring.

The electrochemistry of berberine in acetonitrile is complex and neither the one-electron oxidation nor the reduction produces chemically reversible cyclic voltammetric waves. The electrode potential for the oxidation was estimated from fast scan cyclic voltammetry and that for the reduction from steady-state microelectrode voltammetry. The difference between these potentials, the electrochemical gap, was 2.64 V in good agreement with DFT calculations employing hybrid functionals. The calculations indicate that the spin density of the one-electron reduction product is localised on the half of the molecule containing the isoquinolium moiety, but that the spin density of the one-electron oxidation product is delocalised over the whole molecule. This suggests that a range of structures are possible if attempts are made to oxidatively copolymerise berberine with other conjugated monomers.

## Author contributions

RMGR, BRH and DV supervised the work, raised funding and designed the study. The isolation, purification, electrochemical and optical characterisation and ^1^H NMR simulation was carried out by AUM, HMNPG, MGSAMEWDDKE, JMSJ, UR, WHMRNKH, LS, SW, PGPRA, VNS. HMNPG and LLKP drew the graphical abstract. Microelectrode measurements and TD-DFT simulations were performed by BRH. The manuscript was prepared by RMGR, BRH and HMNPG. All coauthors edited and approved the manuscript.

## Conflicts of interest

There are no conflicts to declare.

## Supplementary Material

RA-013-D3RA01769A-s001
