# Supplementary material for: Berberine isolation from Coscinium fenestratum: optical, electrochemical, and computational studies
Source: RSC Adv. 2023 Jun 7;13(25):17062–73. doi: 10.1039/d3ra01769a (PMC10245225; doi:10.1039/d3ra01769a)
Supplement: RA-013-D3RA01769A-s001 [file RA-013-D3RA01769A-s001.pdf]

Electronic Supplementary Material (ESI) for RSC Advances.
This journal is © The Royal Society of Chemistry 2023

Supporting Information for:

Berberine isolation from *Coscinium fenestratum*: optical, electrochemical, and computational studies

R. M. Gamini Rajapakse [a], Benjamin R. Horrocks [b*], A. U. Malikaramage [a], H. M. N. P. Gunarathna [a], M. G. S. A. M. E. W. D. D. K. Egodawele [a], J.M. Susanthi Jayasinghe [a], Udayana Ranatunga [a], W. H. M. R. N. K. Herath [a], Lahiru Sandakelum [a], Shane Wylie [a], P. G. P. R. Abewardana [a], V.N. Seneviratne [a], L.L.K. Perera [a] and D. Velauthapillai [c]*

[a] *Department of Chemistry, University of Peradeniya, Peradeniya 20400, Sri Lanka*
[b] *School of Natural and Environmental Sciences, Newcastle University, Newcastle upon Tyne NE1 4LB, United Kingdom*
[c] *Advanced Nanomaterials for Clean Energy and Health Applications, Faculty of Engineering and Science, Campus Bergen, Western Norway University of Applied Sciences, Bergen, KRONSTAD, D412, Norway*

Contents

TD-DFT calculations of absorption spectra – additional detail (S1)

FTIR Spectra of isolated and commercial berberine (S2)

Electron micrographs of isolated berberine (S3)

Energy-dispersive analysis of X-rays – mapping and spectra (S4)

1. TD-DFT calculations of absorption spectra – additional detail

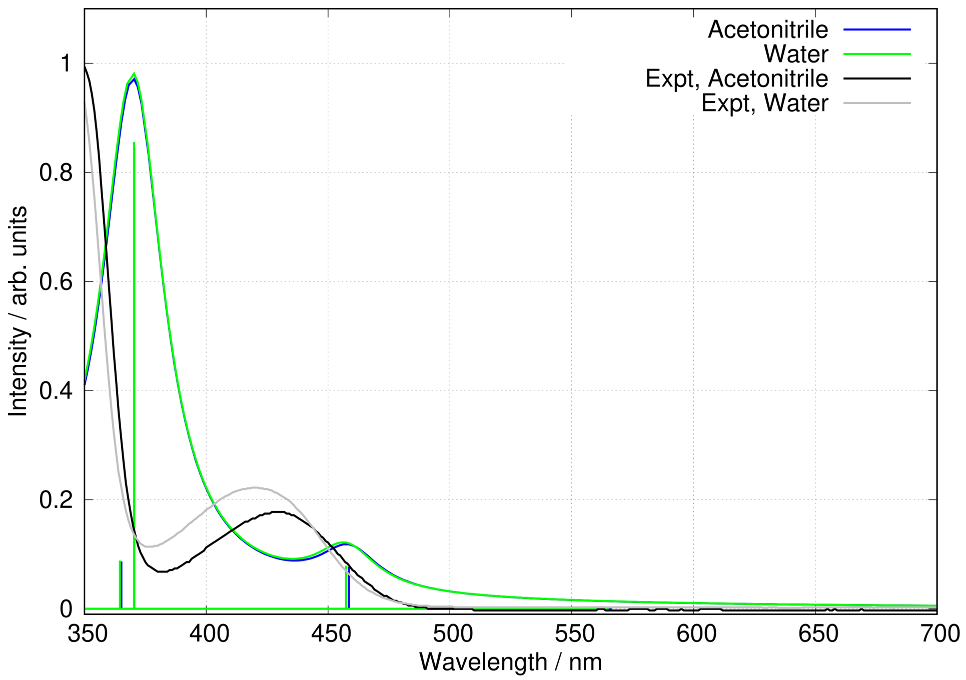

Figure S1: Simulated absorption spectra of the berberine cation in water, acetonitrile, and vacuum. The model chemistry was B3LYP/6-31G(d). The figure presents a zoomed-in region of the spectrum (Fig. 7. main manuscript) showing the $S_0 \rightarrow S_1$ transition more clearly.

2. FTIR Spectra of isolated and commercial berberine

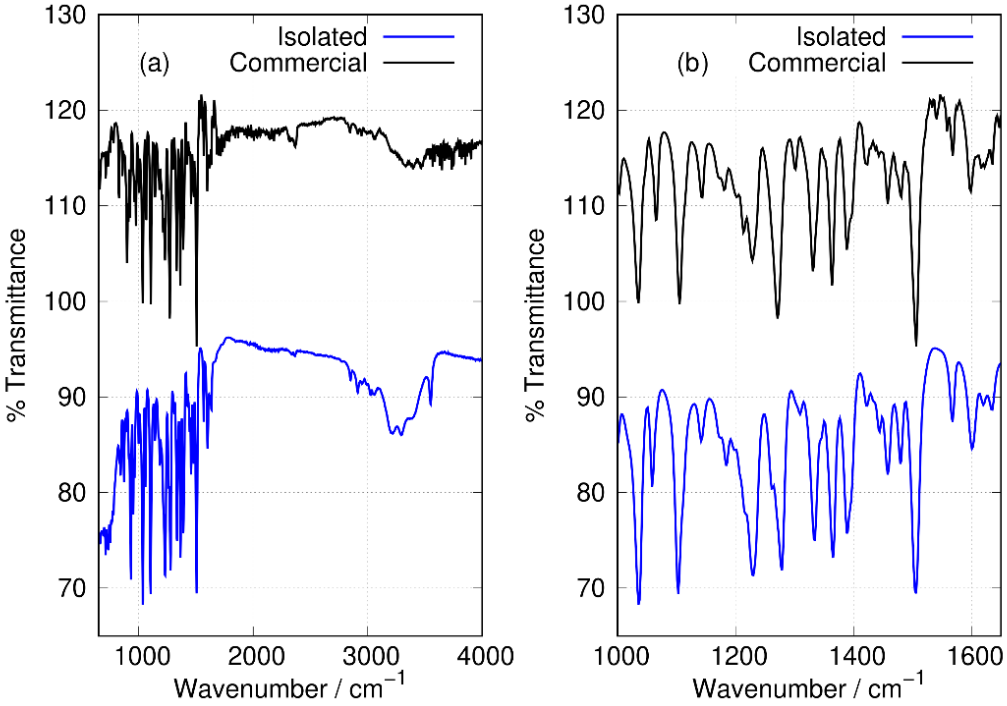

Figure S2: *(a) FTIR spectra of the purified, isolated product and of a commercial sample of berberine chloride. (b) The fingerprint region of the FTIR spectra. The resolution was 4 cm^{-1} in both cases and the spectra are offset on the y-axis for clarity.*

3. SEM Images of crystals of the isolated berberine chloride

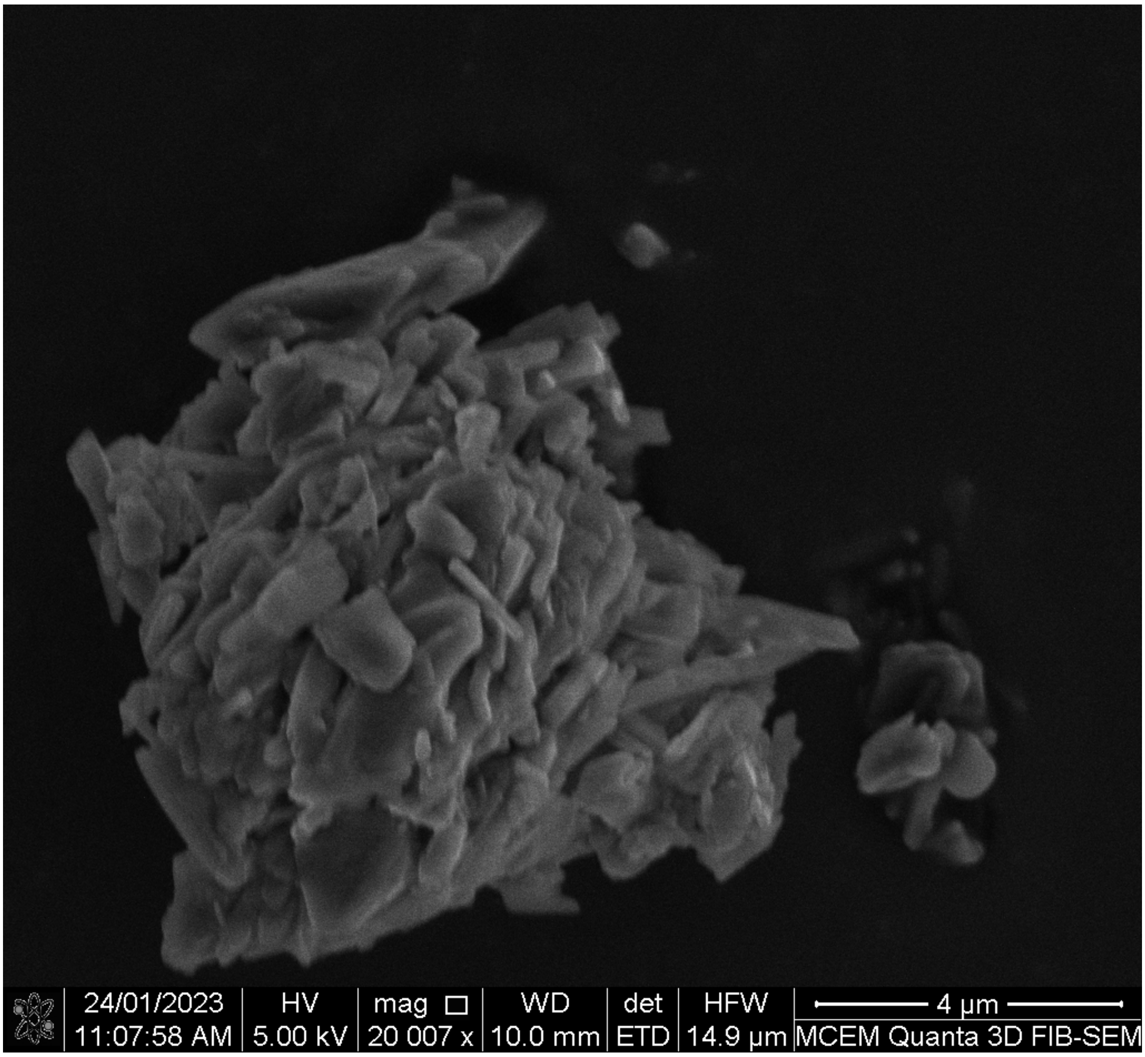

Figure S3: *Electron micrograph of crystals of berberine chloride isolated from Coscinium fenestratum.*

4. Energy dispersive X-ray Analysis of the isolated berberine chloride

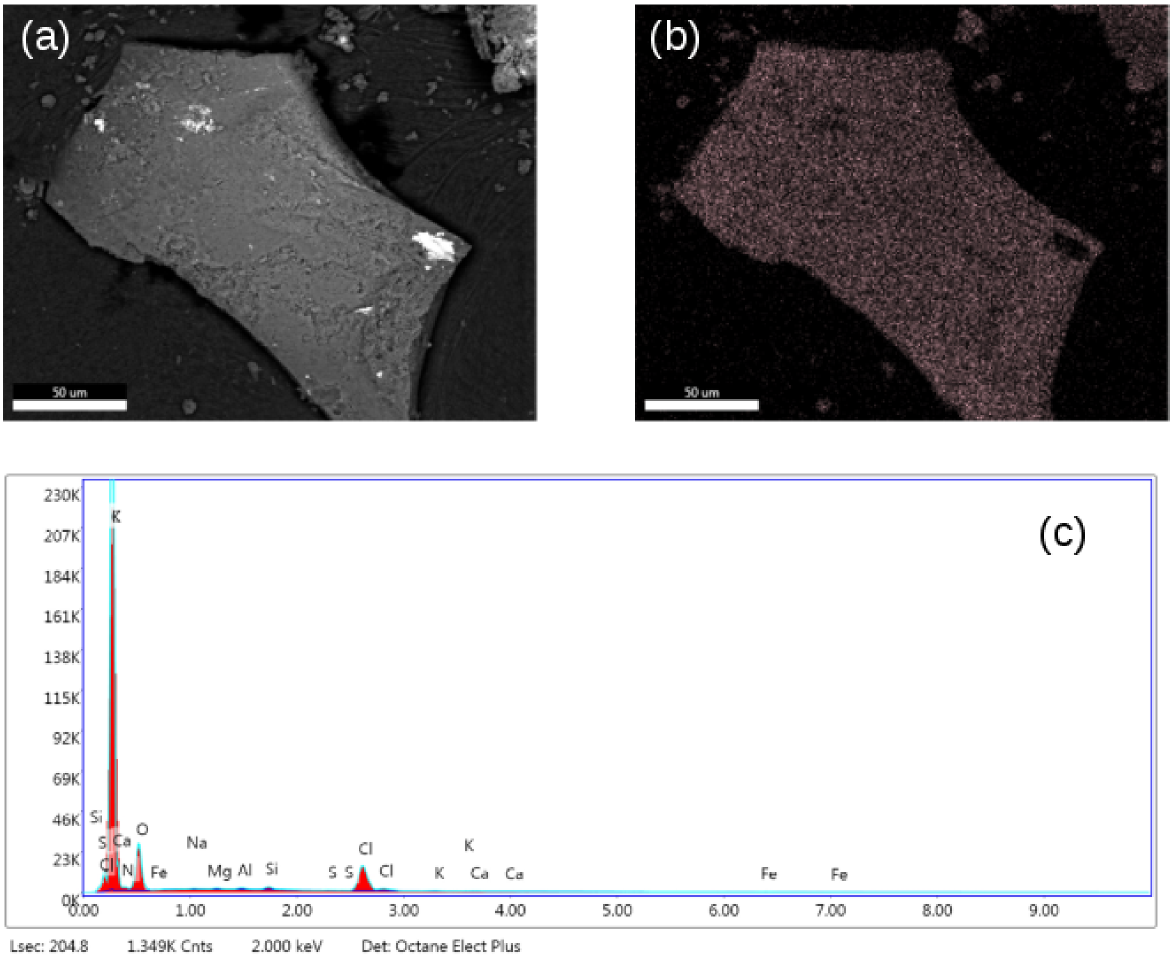

Figure S4: *Electron microscopy and energy-dispersive X-ray spectra of crystals of berberine chloride isolated from Coscinium fenestratum. (a) Electron micrograph of a crystallite. (b) False colour EDX map of Cl Ka and (c) EDX spectrum, Intensity / kcounts against energy. In (a) and (b) the scale bar represents 50 μm.*

These maps and spectra confirm the presence of chloride as the counteranion in the isolated berberine samples.
